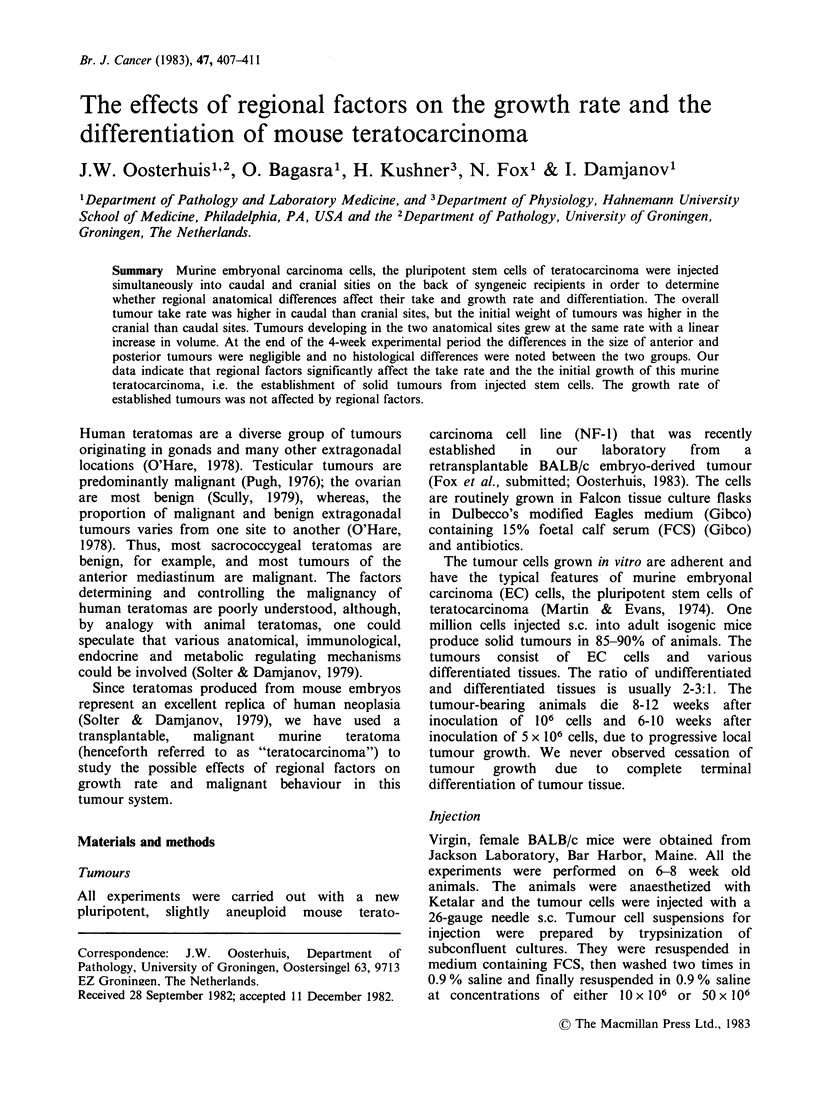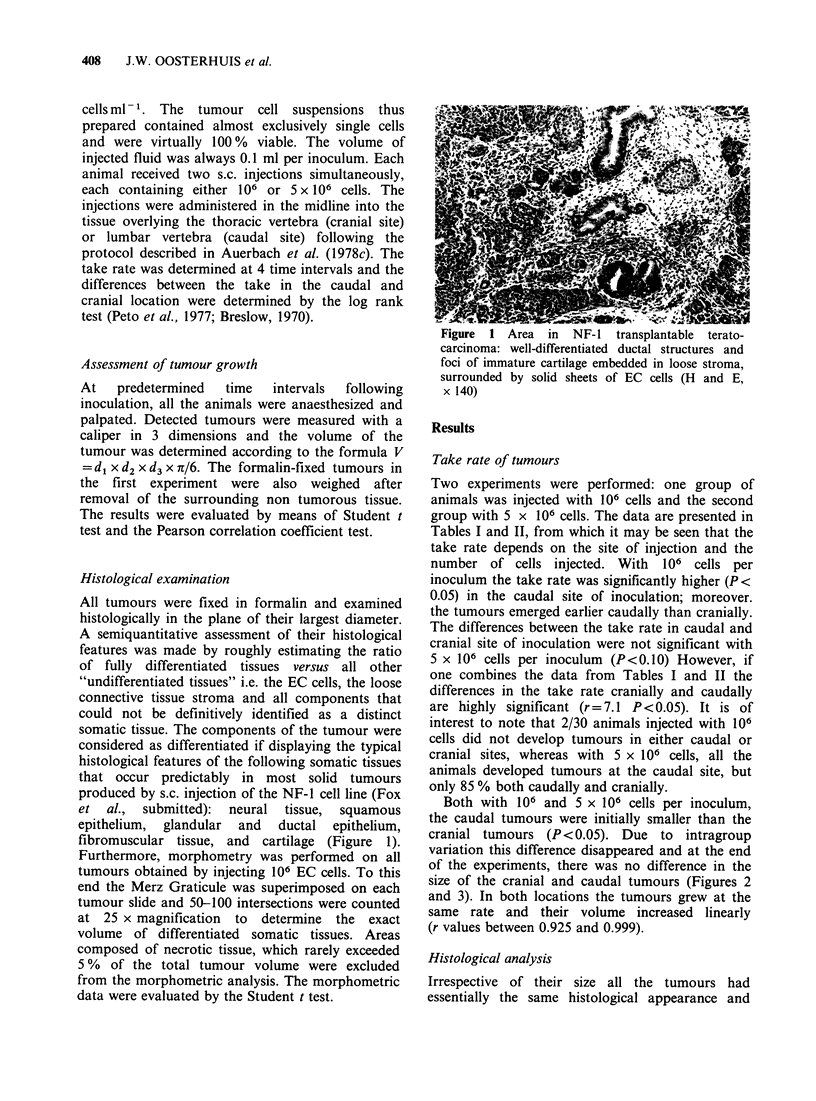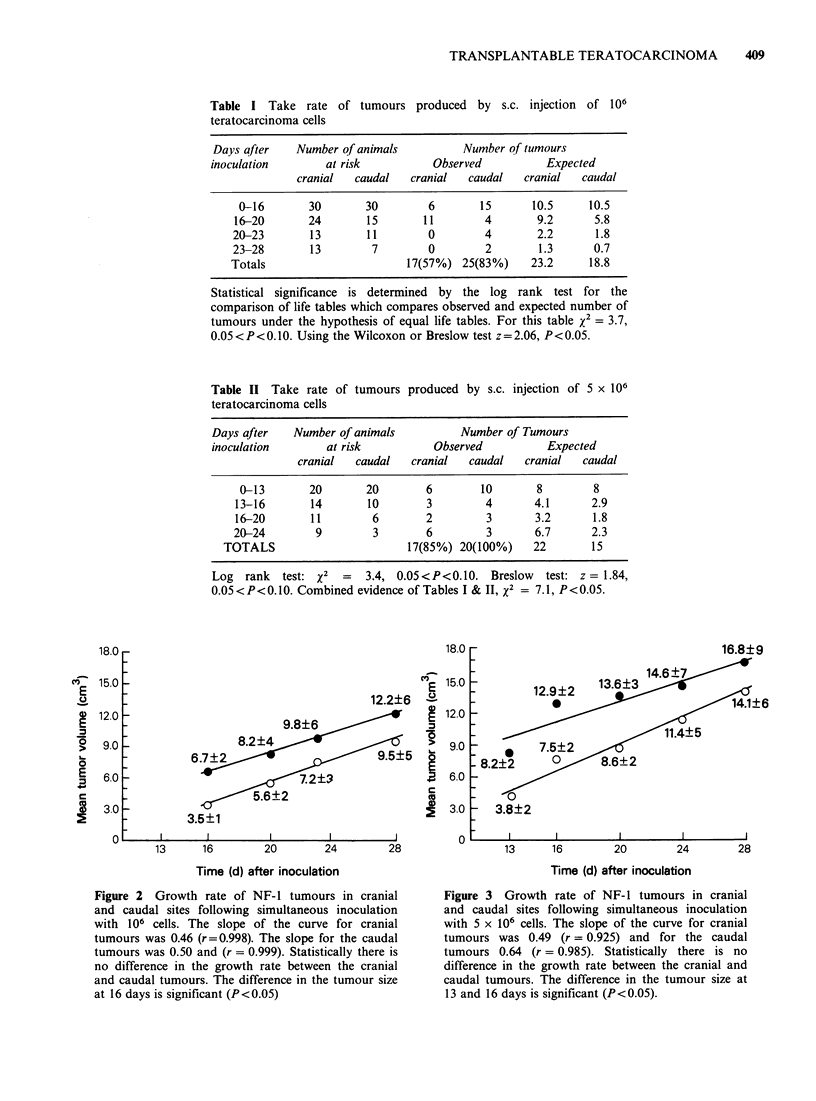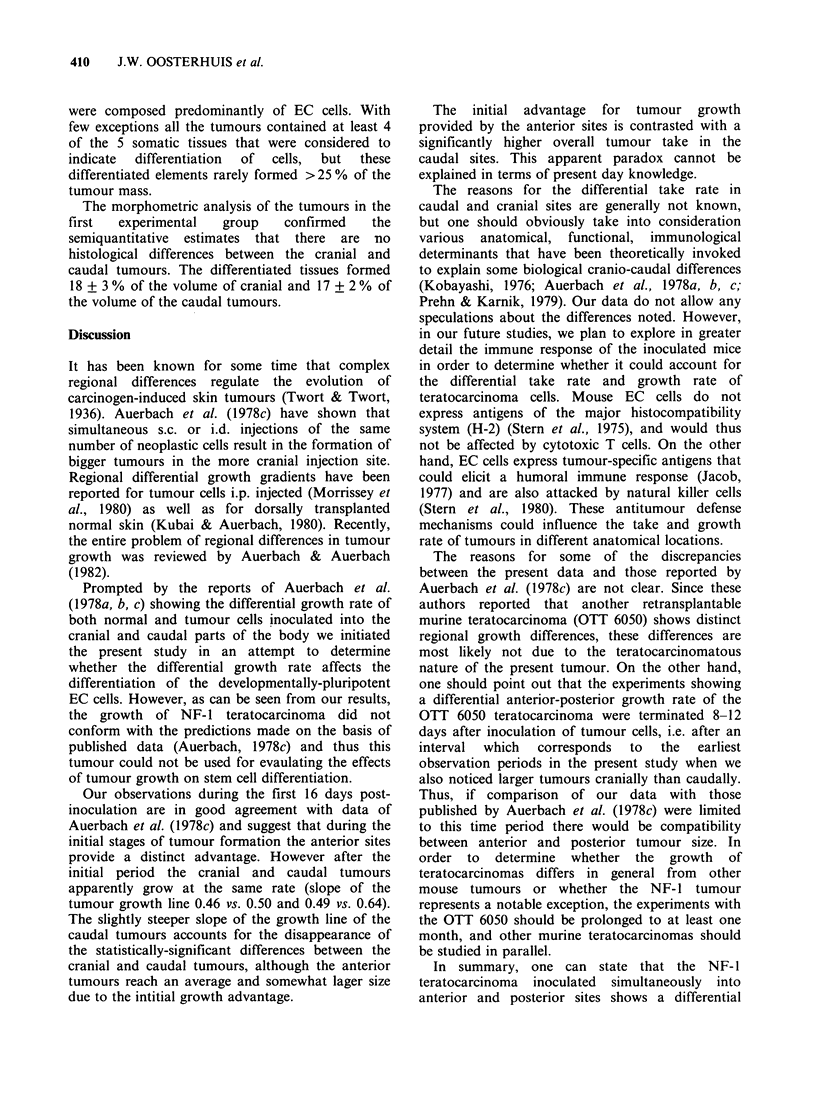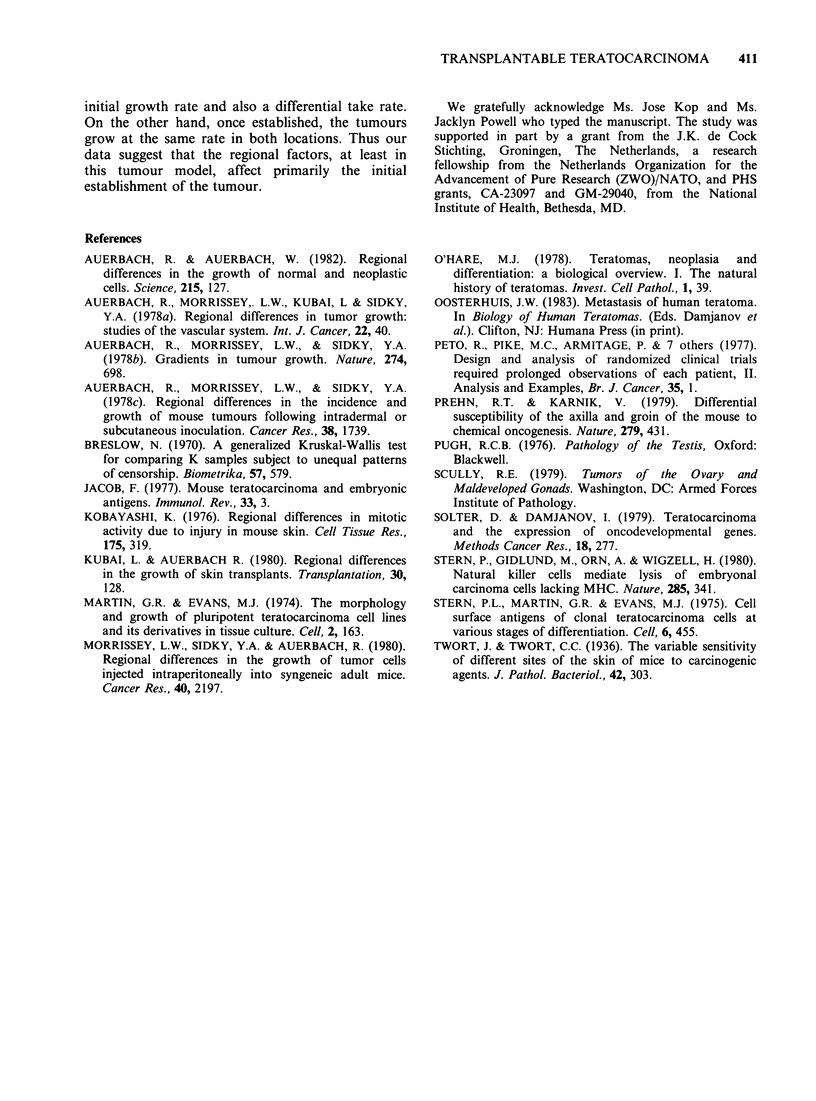# The effects of regional factors on the growth rate and the differentiation of mouse teratocarcinoma.

**DOI:** 10.1038/bjc.1983.61

**Published:** 1983-03

**Authors:** J. W. Oosterhuis, O. Bagasra, H. Kushner, N. Fox, I. Damjanov

## Abstract

**Images:**


Br. J. Cancer (1983), 47, 407–411

### The effects of regional factors on the growth rate and the differentiation of mouse teratocarcinoma

J.W. Oosterhuis[1,2], O. Bagasra[1], H. Kushner[3], N. Fox[1] & I. Damjanov[1]

[1]*Department of Pathology and Laboratory Medicine, and* [3]*Department of Physiology, Hahnemann University School of Medicine, Philadelphia, PA, USA and the* [2]*Department of Pathology, University of Groningen, Groningen, The Netherlands.*

**Summary** Murine embryonal carcinoma cells, the pluripotent stem cells of teratocarcinoma were injected simultaneously into caudal and cranial sities on the back of syngeneic recipients in order to determine whether regional anatomical differences affect their take and growth rate and differentiation. The overall tumour take rate was higher in caudal than cranial sites, but the initial weight of tumours was higher in the cranial than caudal sites. Tumours developing in the two anatomical sites grew at the same rate with a linear increase in volume. At the end of the 4-week experimental period the differences in the size of anterior and posterior tumours were negligible and no histological differences were noted between the two groups. Our data indicate that regional factors significantly affect the take rate and the the initial growth of this murine teratocarcinoma, i.e. the establishment of solid tumours from injected stem cells. The growth rate of established tumours was not affected by regional factors.

Human teratomas are a diverse group of tumours originating in gonads and many other extragonadal locations (O'Hare, 1978). Testicular tumours are predominantly malignant (Pugh, 1976); the ovarian are most benign (Scully, 1979), whereas, the proportion of malignant and benign extragonadal tumours varies from one site to another (O'Hare, 1978). Thus, most sacrococcygeal teratomas are benign, for example, and most tumours of the anterior mediastinum are malignant. The factors determining and controlling the malignancy of human teratomas are poorly understood, although, by analogy with animal teratomas, one could speculate that various anatomical, immunological, endocrine and metabolic regulating mechanisms could be involved (Solter & Damjanov, 1979).

Since teratomas produced from mouse embryos represent an excellent replica of human neoplasia (Solter & Damjanov, 1979), we have used a transplantable, malignant murine teratoma (henceforth referred to as "teratocarcinoma") to study the possible effects of regional factors on growth rate and malignant behaviour in this tumour system.

### Materials and methods

### *Tumours*

All experiments were carried out with a new pluripotent, slightly aneuploid mouse teratocarcinoma cell line (NF-1) that was recently established in our laboratory from a retransplantable BALB/c embryo-derived tumour (Fox *et al.*, submitted; Oosterhuis, 1983). The cells are routinely grown in Falcon tissue culture flasks in Dulbecco's modified Eagles medium (Gibco) containing 15% foetal calf serum (FCS) (Gibco) and antibiotics.

The tumour cells grown *in vitro* are adherent and have the typical features of murine embryonal carcinoma (EC) cells, the pluripotent stem cells of teratocarcinoma (Martin & Evans, 1974). One million cells injected s.c. into adult isogenic mice produce solid tumours in 85–90% of animals. The tumours consist of EC cells and various differentiated tissues. The ratio of undifferentiated and differentiated tissues is usually 2-3:1. The tumour-bearing animals die 8-12 weeks after inoculation of $10^6$ cells and 6-10 weeks after inoculation of $5 \times 10^6$ cells, due to progressive local tumour growth. We never observed cessation of tumour growth due to complete terminal differentiation of tumour tissue.

### *Injection*

Virgin, female BALB/c mice were obtained from Jackson Laboratory, Bar Harbor, Maine. All the experiments were performed on 6–8 week old animals. The animals were anaesthetized with Ketalar and the tumour cells were injected with a 26-gauge needle s.c. Tumour cell suspensions for injection were prepared by trypsinization of subconfluent cultures. They were resuspended in medium containing FCS, then washed two times in 0.9% saline and finally resuspended in 0.9% saline at concentrations of either $10 \times 10^6$ or $50 \times 10^6$

Correspondence: J.W. Oosterhuis, Department of Pathology, University of Groningen, Oostersingel 63, 9713 EZ Groningen, The Netherlands.
Received 28 September 1982; accepted 11 December 1982.

© The Macmillan Press Ltd., 1983

cells $ml^{-1}$. The tumour cell suspensions thus prepared contained almost exclusively single cells and were virtually 100% viable. The volume of injected fluid was always 0.1 ml per inoculum. Each animal received two s.c. injections simultaneously, each containing either $10^6$ or $5 \times 10^6$ cells. The injections were administered in the midline into the tissue overlying the thoracic vertebra (cranial site) or lumbar vertebra (caudal site) following the protocol described in Auerbach *et al.* (1978*c*). The take rate was determined at 4 time intervals and the differences between the take in the caudal and cranial location were determined by the log rank test (Peto *et al.*, 1977; Breslow, 1970).

*Assessment of tumour growth*

At predetermined time intervals following inoculation, all the animals were anaesthesized and palpated. Detected tumours were measured with a caliper in 3 dimensions and the volume of the tumour was determined according to the formula $V = d_1 \times d_2 \times d_3 \times \pi/6$. The formalin-fixed tumours in the first experiment were also weighed after removal of the surrounding non tumorous tissue. The results were evaluated by means of Student $t$ test and the Pearson correlation coefficient test.

*Histological examination*

All tumours were fixed in formalin and examined histologically in the plane of their largest diameter. A semiquantitative assessment of their histological features was made by roughly estimating the ratio of fully differentiated tissues *versus* all other "undifferentiated tissues" i.e. the EC cells, the loose connective tissue stroma and all components that could not be definitively identified as a distinct somatic tissue. The components of the tumour were considered as differentiated if displaying the typical histological features of the following somatic tissues that occur predictably in most solid tumours produced by s.c. injection of the NF-1 cell line (Fox *et al.*, submitted): neural tissue, squamous epithelium, glandular and ductal epithelium, fibromuscular tissue, and cartilage (Figure 1). Furthermore, morphometry was performed on all tumours obtained by injecting $10^6$ EC cells. To this end the Merz Graticule was superimposed on each tumour slide and 50–100 intersections were counted at 25 × magnification to determine the exact volume of differentiated somatic tissues. Areas composed of necrotic tissue, which rarely exceeded 5% of the total tumour volume were excluded from the morphometric analysis. The morphometric data were evaluated by the Student $t$ test.

**Figure 1** Area in NF-1 transplantable teratocarcinoma: well-differentiated ductal structures and foci of immature cartilage embedded in loose stroma, surrounded by solid sheets of EC cells (H and E, × 140)

### Results

*Take rate of tumours*

Two experiments were performed: one group of animals was injected with $10^6$ cells and the second group with $5 \times 10^6$ cells. The data are presented in Tables I and II, from which it may be seen that the take rate depends on the site of injection and the number of cells injected. With $10^6$ cells per inoculum the take rate was significantly higher ($P < 0.05$) in the caudal site of inoculation; moreover. the tumours emerged earlier caudally than cranially. The differences between the take rate in caudal and cranial site of inoculation were not significant with $5 \times 10^6$ cells per inoculum ($P < 0.10$) However, if one combines the data from Tables I and II the differences in the take rate cranially and caudally are highly significant ($r = 7.1$ $P < 0.05$). It is of interest to note that 2/30 animals injected with $10^6$ cells did not develop tumours in either caudal or cranial sites, whereas with $5 \times 10^6$ cells, all the animals developed tumours at the caudal site, but only 85% both caudally and cranially.

Both with $10^6$ and $5 \times 10^6$ cells per inoculum, the caudal tumours were initially smaller than the cranial tumours ($P < 0.05$). Due to intragroup variation this difference disappeared and at the end of the experiments, there was no difference in the size of the cranial and caudal tumours (Figures 2 and 3). In both locations the tumours grew at the same rate and their volume increased linearly ($r$ values between 0.925 and 0.999).

*Histological analysis*

Irrespective of their size all the tumours had essentially the same histological appearance and

**Table I** Take rate of tumours produced by s.c. injection of $10^6$ teratocarcinoma cells

| *Days after inoculation* | *Number of animals at risk* | | *Number of tumours* | | | |
|---|---|---|---|---|---|---|
| | | | *Observed* | | *Expected* | |
| | *cranial* | *caudal* | *cranial* | *caudal* | *cranial* | *caudal* |
| 0–16 | 30 | 30 | 6 | 15 | 10.5 | 10.5 |
| 16–20 | 24 | 15 | 11 | 4 | 9.2 | 5.8 |
| 20–23 | 13 | 11 | 0 | 4 | 2.2 | 1.8 |
| 23–28 | 13 | 7 | 0 | 2 | 1.3 | 0.7 |
| Totals | | | 17(57%) | 25(83%) | 23.2 | 18.8 |

Statistical significance is determined by the log rank test for the comparison of life tables which compares observed and expected number of tumours under the hypothesis of equal life tables. For this table $\chi^2 = 3.7$, $0.05<P<0.10$. Using the Wilcoxon or Breslow test $z=2.06$, $P<0.05$.

**Table II** Take rate of tumours produced by s.c. injection of $5 \times 10^6$ teratocarcinoma cells

| *Days after inoculation* | *Number of animals at risk* | | *Number of Tumours* | | | |
|---|---|---|---|---|---|---|
| | | | *Observed* | | *Expected* | |
| | *cranial* | *caudal* | *cranial* | *caudal* | *cranial* | *caudal* |
| 0–13 | 20 | 20 | 6 | 10 | 8 | 8 |
| 13–16 | 14 | 10 | 3 | 4 | 4.1 | 2.9 |
| 16–20 | 11 | 6 | 2 | 3 | 3.2 | 1.8 |
| 20–24 | 9 | 3 | 6 | 3 | 6.7 | 2.3 |
| TOTALS | | | 17(85%) | 20(100%) | 22 | 15 |

Log rank test: $\chi^2 = 3.4$, $0.05<P<0.10$. Breslow test: $z = 1.84$, $0.05<P<0.10$. Combined evidence of Tables I & II, $\chi^2 = 7.1$, $P<0.05$.

**Figure 2** Growth rate of NF-1 tumours in cranial and caudal sites following simultaneous inoculation with $10^6$ cells. The slope of the curve for cranial tumours was 0.46 ($r=0.998$). The slope for the caudal tumours was 0.50 and ($r = 0.999$). Statistically there is no difference in the growth rate between the cranial and caudal tumours. The difference in the tumour size at 16 days is significant ($P<0.05$)

**Figure 3** Growth rate of NF-1 tumours in cranial and caudal sites following simultaneous inoculation with $5 \times 10^6$ cells. The slope of the curve for cranial tumours was 0.49 ($r = 0.925$) and for the caudal tumours 0.64 ($r = 0.985$). Statistically there is no difference in the growth rate between the cranial and caudal tumours. The difference in the tumour size at 13 and 16 days is significant ($P<0.05$).

were composed predominantly of EC cells. With few exceptions all the tumours contained at least 4 of the 5 somatic tissues that were considered to indicate differentiation of cells, but these differentiated elements rarely formed >25% of the tumour mass.

The morphometric analysis of the tumours in the first experimental group confirmed the semiquantitative estimates that there are no histological differences between the cranial and caudal tumours. The differentiated tissues formed $18 \pm 3\%$ of the volume of cranial and $17 \pm 2\%$ of the volume of the caudal tumours.

### Discussion

It has been known for some time that complex regional differences regulate the evolution of carcinogen-induced skin tumours (Twort & Twort, 1936). Auerbach *et al.* (1978*c*) have shown that simultaneous s.c. or i.d. injections of the same number of neoplastic cells result in the formation of bigger tumours in the more cranial injection site. Regional differential growth gradients have been reported for tumour cells i.p. injected (Morrissey *et al.*, 1980) as well as for dorsally transplanted normal skin (Kubai & Auerbach, 1980). Recently, the entire problem of regional differences in tumour growth was reviewed by Auerbach & Auerbach (1982).

Prompted by the reports of Auerbach *et al.* (1978*a, b, c*) showing the differential growth rate of both normal and tumour cells inoculated into the cranial and caudal parts of the body we initiated the present study in an attempt to determine whether the differential growth rate affects the differentiation of the developmentally-pluripotent EC cells. However, as can be seen from our results, the growth of NF-1 teratocarcinoma did not conform with the predictions made on the basis of published data (Auerbach, 1978*c*) and thus this tumour could not be used for evaulating the effects of tumour growth on stem cell differentiation.

Our observations during the first 16 days post-inoculation are in good agreement with data of Auerbach *et al.* (1978*c*) and suggest that during the initial stages of tumour formation the anterior sites provide a distinct advantage. However after the initial period the cranial and caudal tumours apparently grow at the same rate (slope of the tumour growth line 0.46 *vs.* 0.50 and 0.49 *vs.* 0.64). The slightly steeper slope of the growth line of the caudal tumours accounts for the disappearance of the statistically-significant differences between the cranial and caudal tumours, although the anterior tumours reach an average and somewhat lager size due to the intitial growth advantage.

The initial advantage for tumour growth provided by the anterior sites is contrasted with a significantly higher overall tumour take in the caudal sites. This apparent paradox cannot be explained in terms of present day knowledge.

The reasons for the differential take rate in caudal and cranial sites are generally not known, but one should obviously take into consideration various anatomical, functional, immunological determinants that have been theoretically invoked to explain some biological cranio-caudal differences (Kobayashi, 1976; Auerbach *et al.*, 1978*a, b, c;* Prehn & Karnik, 1979). Our data do not allow any speculations about the differences noted. However, in our future studies, we plan to explore in greater detail the immune response of the inoculated mice in order to determine whether it could account for the differential take rate and growth rate of teratocarcinoma cells. Mouse EC cells do not express antigens of the major histocompatibility system (H-2) (Stern *et al.*, 1975), and would thus not be affected by cytotoxic T cells. On the other hand, EC cells express tumour-specific antigens that could elicit a humoral immune response (Jacob, 1977) and are also attacked by natural killer cells (Stern *et al.*, 1980). These antitumour defense mechanisms could influence the take and growth rate of tumours in different anatomical locations.

The reasons for some of the discrepancies between the present data and those reported by Auerbach *et al.* (1978*c*) are not clear. Since these authors reported that another retransplantable murine teratocarcinoma (OTT 6050) shows distinct regional growth differences, these differences are most likely not due to the teratocarcinomatous nature of the present tumour. On the other hand, one should point out that the experiments showing a differential anterior-posterior growth rate of the OTT 6050 teratocarcinoma were terminated 8–12 days after inoculation of tumour cells, i.e. after an interval which corresponds to the earliest observation periods in the present study when we also noticed larger tumours cranially than caudally. Thus, if comparison of our data with those published by Auerbach *et al.* (1978*c*) were limited to this time period there would be compatibility between anterior and posterior tumour size. In order to determine whether the growth of teratocarcinomas differs in general from other mouse tumours or whether the NF-1 tumour represents a notable exception, the experiments with the OTT 6050 should be prolonged to at least one month, and other murine teratocarcinomas should be studied in parallel.

In summary, one can state that the NF-1 teratocarcinoma inoculated simultaneously into anterior and posterior sites shows a differential initial growth rate and also a differential take rate. On the other hand, once established, the tumours grow at the same rate in both locations. Thus our data suggest that the regional factors, at least in this tumour model, affect primarily the initial establishment of the tumour.

We gratefully acknowledge Ms. Jose Kop and Ms. Jacklyn Powell who typed the manuscript. The study was supported in part by a grant from the J.K. de Cock Stichting, Groningen, The Netherlands, a research fellowship from the Netherlands Organization for the Advancement of Pure Research (ZWO)/NATO, and PHS grants, CA-23097 and GM-29040, from the National Institute of Health, Bethesda, MD.

### References

AUERBACH, R. & AUERBACH, W. (1982). Regional differences in the growth of normal and neoplastic cells. *Science*, **215**, 127.

AUERBACH, R., MORRISSEY,. L.W., KUBAI, L & SIDKY, Y.A. (1978*a*). Regional differences in tumor growth: studies of the vascular system. *Int. J. Cancer*, **22**, 40.

AUERBACH, R., MORRISSEY, L.W., & SIDKY, Y.A. (1978*b*). Gradients in tumour growth. *Nature*, **274**, 698.

AUERBACH, R., MORRISSEY, L.W., & SIDKY, Y.A. (1978*c*). Regional differences in the incidence and growth of mouse tumours following intradermal or subcutaneous inoculation. *Cancer Res.*, **38**, 1739.

BRESLOW, N. (1970). A generalized Kruskal-Wallis test for comparing K samples subject to unequal patterns of censorship. *Biometrika*, **57**, 579.

JACOB, F. (1977). Mouse teratocarcinoma and embryonic antigens. *Immunol. Rev.*, **33**, 3.

KOBAYASHI, K. (1976). Regional differences in mitotic activity due to injury in mouse skin. *Cell Tissue Res.*, **175**, 319.

KUBAI, L. & AUERBACH R. (1980). Regional differences in the growth of skin transplants. *Transplantation*, **30**, 128.

MARTIN, G.R. & EVANS, M.J. (1974). The morphology and growth of pluripotent teratocarcinoma cell lines and its derivatives in tissue culture. *Cell*, **2**, 163.

MORRISSEY, L.W., SIDKY, Y.A. & AUERBACH, R. (1980). Regional differences in the growth of tumor cells injected intraperitoneally into syngeneic adult mice. *Cancer Res.*, **40**, 2197.

O'HARE, M.J. (1978). Teratomas, neoplasia and differentiation: a biological overview. I. The natural history of teratomas. *Invest. Cell Pathol.*, **1**, 39.

OOSTERHUIS, J.W. (1983). Metastasis of human teratoma. In *Biology of Human Teratomas*. (Eds. Damjanov *et al.*). Clifton, NJ: Humana Press (in print).

PETO, R., PIKE, M.C., ARMITAGE, P. & 7 others (1977). Design and analysis of randomized clinical trials required prolonged observations of each patient, II. Analysis and Examples, *Br. J. Cancer*, **35**, 1.

PREHN, R.T. & KARNIK, V. (1979). Differential susceptibility of the axilla and groin of the mouse to chemical oncogenesis. *Nature*, **279**, 431.

PUGH, R.C.B. (1976). *Pathology of the Testis*, Oxford: Blackwell.

SCULLY, R.E. (1979). *Tumors of the Ovary and Maldeveloped Gonads*. Washington, DC: Armed Forces Institute of Pathology.

SOLTER, D. & DAMJANOV, I. (1979). Teratocarcinoma and the expression of oncodevelopmental genes. *Methods Cancer Res.*, **18**, 277.

STERN, P., GIDLUND, M., ORN, A. & WIGZELL, H. (1980). Natural killer cells mediate lysis of embryonal carcinoma cells lacking MHC. *Nature*, **285**, 341.

STERN, P.L., MARTIN, G.R. & EVANS, M.J. (1975). Cell surface antigens of clonal teratocarcinoma cells at various stages of differentiation. *Cell*, **6**, 455.

TWORT, J. & TWORT, C.C. (1936). The variable sensitivity of different sites of the skin of mice to carcinogenic agents. *J. Pathol. Bacteriol.*, **42**, 303.